# Hormesis: Decoding Two Sides of the Same Coin

**DOI:** 10.3390/ph8040865

**Published:** 2015-12-16

**Authors:** Dipita Bhakta-Guha, Thomas Efferth

**Affiliations:** 1 School of Chemical and Biotechnology, SASTRA University, Thanjavur, Tamil Nadu 613401, India, E-Mail: dipita2001@gmail.com; 2 Department of Pharmaceutical Biology, Johannes Gutenberg University, Mainz 55128, Germany

**Keywords:** hormesis, biphasic, cancer, pathways, stressor, mimetics

## Abstract

In the paradigm of drug administration, determining the correct dosage of a therapeutic is often a challenge. Several drugs have been noted to demonstrate contradictory effects *per se* at high and low doses. This duality in function of a drug at different concentrations is known as hormesis. Therefore, it becomes necessary to study these biphasic functions in order to understand the mechanistic basis of their effects. In this article, we focus on different molecules and pathways associated with diseases that possess a duality in their function and thus prove to be the seat of hormesis. In particular, we have highlighted the pathways and factors involved in the progression of cancer and how the biphasic behavior of the molecules involved can alter the manifestations of cancer. Because of the pragmatic role that it exhibits, the imminent need is to draw attention to the concept of hormesis. Herein, we also discuss different stressors that trigger hormesis and how stress-mediated responses increase the overall adaptive response of an individual to stress stimulus. We talk about common pathways through which cancer progresses (such as nuclear factor erythroid 2-related factor 2-Kelch-like ECH-associated protein 1 (Nrf2-Keap1), sirtuin-forkhead box O (SIRT-FOXO) and others), analyzing how diverse molecules associated with these pathways conform to hormesis.

## 1. Introduction

For several decades, it was believed that drug dosage follows a linear pattern, generating enormous ignorance in the knowledge of the responses in the low-dose zone [1]. Nonetheless, in the past few years, several studies have depicted an inverse response to varied drug doses in the same individual, thereby thoroughly discarding the linearity and threshold-response models of dose determination [2,3]. This reaction, known as “biphasic dose response”, as revealed by several studies, has shown significance in establishing the modality of a drug [4,5,6,7]. This phenomenon determines the optimal dose of a prescribed drug and, thus, very well advocates Philip von Hohenheim’s adage “*Die Dosis macht das Gift*” or “the dose makes the poison” [8].

**Figure 1:**
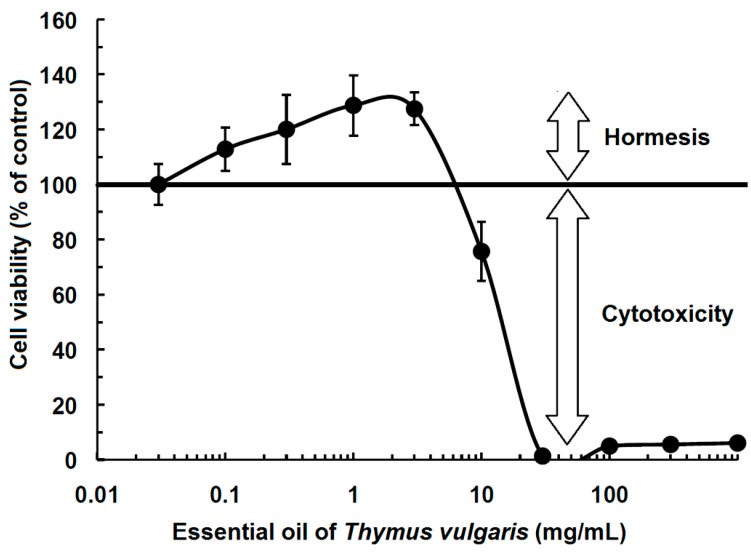
Hormetic effect of essential oil obtained from *Thymus vulgaris* cell viability (taken and modified with permission of the International Institute of Anticancer Research, Greece). Human UMSCC1 squamous cell carcinoma cells were treated with and without essential oil of Thymus vulgaris and subjected to transcriptome-wide microarray analyses. A total number of 804 genes were differentially regulated in treated cells compared to untreated control cells. These were subjected to ingenuity pathway analysis (IPA) profiling to identify possible pathways. The top three pathways that contribute to the mode of action of the essential oil were: (1) interferon signaling, (2) *N*-glycan biosynthesis and (3) ERK5 signaling [16].

It is well recorded that mild environmental stress as a result of feeble doses of stressor stimuli often incites adaptive stress response in individuals in order to maintain homeostasis [9,10]. It also complies with the fact that while higher doses of an insult can be overtly harmful, small doses of the same can actually promote health, governed by growth and development [4,11]. For instance, we all know that oxygen is indispensable for all aerobic life forms. However, it is interesting to note that the same elixir at high concentrations can prove to be toxic, thus establishing that oxygen follows a biphasic dose relationship [12]. Hence, oxygen is considered beneficial at environmental doses and fatal at higher concentrations [13,14]. Such a biphasic response brings into light an immensely significant concept called “hormesis”, which refers to a phenomenon that is designated by a “low-dose stimulation and high-dose inhibition” [3]. In other words, hormesis is defined as the duality in response by a cell/individual in reply to an impetus (may be endogenous or exogenous) that spurs favorable effects at a low dose and harmful effects at higher measures [15] (Figure 1). Therefore, in contrast to threshold or linear models of dose-response, hormesis is usually represented either by an inverted U- (bell-shaped) or a J-/U-shaped dose-response curve.

Hormesis, in general, might also be elaborated as an adaptive mechanism of a cell or an organism to compensate any imbalance in homeostasis caused by exposure to factors that mediate intermittent mild stresses [10,16,17]. For example, small episodes of mild ischemia referred to as ischemic preconditioning (IPC) have been reported to shield the brain from future severe depletion of blood and, thus, oxygen [10]. In another adaptive biphasic response, mild caloric restriction (CR) in diverse animals was found to be associated with protection against different forms of cancer [18,19,20]. While extreme dietary (calorie) restriction could manifest into malnutrition, mild restriction, on the contrary, evokes an adaptive response that defends an individual from cancer and other progressive degenerative pathologies, such as neuro-degenerative and geriatric diseases [21]. This also takes us to another subcategory of hormesis, ‘mitohormesis’, wherein subjecting an individual to sub-lethal volumes of mitochondrial reactive oxygen species (mtROS) increases the lifespan [22,23]. While ROS in general remains one of the fundamental reasons for most of the diseases, exposure to lower volumes tends to reverse its adversity [24]. It is suggested that ROS might induce endoplasmic reticulum (ER) stress, since a functional axis between ER and mitochondrion exists. This also implies that mitohormesis may be in turn associated with ER hormesis (described later) [25,26]. This biphasic character of hormesis thereby emerges prominently as a subject that needs to be well addressed and understood, especially while determining the dosages of any treatment [3]. Furthermore, it becomes mandatory for the determination of the potential effect of a drug in use at its low-dose zone, so as to comprehend its varied behavior (if it exists). Keeping the duality of responses in mind, regulatory bodies, such as the Food and Drug Administration (FDA) and Environmental Protection Agency (EPA) in the USA, have implemented stringent guidelines to take into account the hormetic effects of interventions [27].

## 2. History

Mithridates VI, the king of Pontus, since childhood, was suspicious that his mother would poison him to death. In a bid to save himself, he regularly ingested small doses of venom under the supervision of the Shamans of Agari, believing, that this would defend him against all poisons [28]. Since then, the system of administering non-lethal doses of venom in an attempt to avert future poisoning has been termed Mithridatism. Although there is no scientific evidence that substantiates the success of Mithridatism, movies, blogs and a few zoo handlers have continued publicizing the practice. On the scientific arena, however, different responses in the same individual as a consequence of varied dosages of the same drug (not necessarily a poison or toxin), intercepted by time, have often been reported.

The term “hormesis” finds its origin in the Greek word “*hormáein*” meaning “to set in motion” [10]. In 1854, Rudolf Virchow reported that beating of tracheal epithelial cilia in postmortem mucosa differed under varied sodium and potassium hydroxide concentrations. While at low concentrations, ciliary beatings increased, a dose-dependent decrease in the activity was observed at higher concentrations [29]. Three decades later, Hugo Schulz observed that low dose application of plausible disinfectants actually enhanced metabolism in yeast, which is diametrically opposite to their activity at higher doses [30]. Although initially he discarded the theory believing the result to be an experimental error, repeated studies encouraged him to postulate the Arndt–Schulz law in association with Rudolf Arndt. In 1943, Southam and Ehrlich presented the term hormesis for the first time in published literature on their work on the effect of red cider tree extract on fungal proliferation [30]. Despite the prominence revealed in several articles in the subsequent years, focus on hormesis began to get diluted primarily owing to the inability to assess the effect of drugs at their low doses. Most of the 20th century witnessed experiments with the high dose response of drugs and failed to comprehend the significance of clinical manifestations of low-dose drug administration. Furthermore, the overall misguided assumption of hormesis being somehow associated only with homeopathy led to a significant loss of interest in the subject.

Around the 1970s, with the emergence of stringent regulatory measures of drug administration along with improvement in analytical and validation methods, interest in hormesis resurfaced [31]. The assessment of the risk of exposure to carcinogens at very low dose gave impetus to the re-evaluation of the concept in particular [32]. Colossal efforts by Calabrese and others re-established the significance of hormesis and made its estimation a mandatory facet in the precinct of dose determination [7,33,34,35,36,37].

## 3. What Triggers Hormesis?

External effectors (stimuli), such as stressors or aggressors that induce stress at higher concentrations are often called hormetins [21,38]. At this juncture, it becomes imperative to establish that the term “stress” in itself can have multiple implications. In the context of this review, we refer to stress as any parameter, extrinsic or intrinsic, that can induce deviation from the normal physiological processes of the body. Exposure to stress often elicits pathways meant to combat the same, which are known as stress-responses [39]. Several of these responses often necessitate stimulation of pro-survival pathways [40]. The hormetins might be of biological, physical or chemical origin [9,41]. The generation of the adaptive response to continuous mild exposures to such stressors is an evolutionarily-conserved trait, which in the long run shields an individual against future high concentration-stressor assaults [10]. Although accurate enumeration of components that trigger hormesis might be difficult, hormetins are primarily of the following types:

### 3.1. Phytochemicals

Compounds derived from plants have been a forerunner in the arena of drug discovery owing to the tremendous potential they harbor in terms of combating diverse diseases [42]. Even though several secondary metabolites at higher concentrations are toxic, administering them at regulated lower doses has demonstrated health benefits [43,44,45,46,47,48,49]. Many of them, especially the dietary phytochemicals (DPCs), have been reported to be prominent hormetic stressors that affect various molecules and signaling pathways associated with disease progression [49]. DPCs, such as chalcone, resveratrol and piceatannol, have exhibited the potential to kill cancer cells at higher doses and simultaneously confer neuro-protection at a low dose administration [43,45,46]. In addition, curcumin, epigallocatechin gallate, genistein, sulforaphane and others specifically affect the Nrf2/antioxidant response element (Nrf2/ARE) pathway [44,47,48].

### 3.2. Temperature

Reaction to heat stress is usually mediated through the heat shock (HS) response pathway, which is an evolutionarily-conserved mechanism [40]. Exposure to heat, in principle, triggers nuclear translocation of HS factors. On binding to DNA, these factors initiate HS gene transcription, thereby leading to the translation of HS proteins (HSPs) [50]. Exposing inbred males of *Drosophila melanogaster* to 37 °C for 5 min daily for a stretch of five days/week has been noted to increase their longevity [51]. In addition, pre-exposing human skin fibroblasts to an hour of HS treatment (41–42 °C) could protect the cells from premature senescence and apoptosis [40].

### 3.3. Caloric Restriction

Aging is no longer considered an outcome of long-term accumulation of molecular damage. Recent studies reveal that aging is a consequence of a hyper-activated target of rapamycin (TOR) pathway that initiates the activation of diverse cellular processes contributing to geroconversion in organisms, ranging from yeast to mammal (mTOR in mammals) [52]. In different animal models, such as mice, *C. elegans*, *D. melanogaster*, *S. cerevisiae*, *etc.*, dietary restriction (reduction of nutrients by limiting food and water supply not amounting to malnutrition) or CR (decline in calories without altering amino acids, vitamins and other nutrients) reportedly deactivates the TOR pathway, while subsequently slowing down the process of aging and, thus, extending longevity [53].

### 3.4. Exercise

According to the stress theory [54], response generated on exposure of the body to a chronic stressor can be primarily split into three phases: decreased alarm, increased resistance and exhaustion. While long-duration exercises (18–24 h continuously) are associated with serious, deleterious exhaustion, a normal exercise regimen actually elicits the adaptive response through controlled moderation of free radicals [55]. Under a normal regimen, each exercise spell is followed by a rest phase, where the body is allowed to cope with the stressor (exercise in this case) and subsequently gets adapted. During regular exercise, ROS-mediated nuclear factor kappa-light chain enhancer of activated B cells (NFκB)-dependent upregulation of superoxide dismutase (SOD) and glutathione peroxidase gene expressions defends against antioxidants [56]. In addition, several genes involved with metabolism and mitochondrial biogenesis (peroxisome proliferator-activated receptor-γ/-δ (PPAR-γ/-δ), IL-6 receptor, forkhead box O 1 (FOXO1)) oxidant stress response (interferon regulatory factor 1 (IRF1), metallothioneins) also get upregulated, thereby augmenting the adaptive response of the body [57].

Hence, hormetins eliciting non-severe mild chronic stress develop an adaptive response that effectively aids in preparing the body against plausible future high concentration stressor insults. Regular exposure to weak doses of stressors leads to an increase in the stress response-mediated somatic maintenance function that in the long term renders cells resistant to stress, subsequently slowing down the rate of aging and cellular degeneration asserting their hormetic role [40]. Mechanistically, a special set of protective/pro-survival genes known as “vitagenes” maintain cellular homeostasis under conditions of stress. These genes, which code for HSPs (such as heme oxygenase-1 (HO-1), Hsp32, Hsp70, Hsp72, sirtuin and thioredoxin protein systems), primarily trigger multiple signaling networks that aid in upholding the adaptive response of a system and help in circumventing the possible fatalities of stress-induced anomalies [58]. Out of the many pathways, hormetic behavior of ROS in aging, bone remodeling and progressive degenerative manifestations is governed by vitagenes [59].

## 4. Hormesis in Stressor-Mediated Pathways

In order to determine how the beneficial role of hormesis might be extrapolated in mitigating malefic consequences of cellular insults, it becomes pertinent to recognize the different cellular/molecular systems and intervening pathways that ensure a biphasic relationship in generating adaptive responses to a stressor stimulus [30]. Primarily, in the context of the etiology of ailments, such as mitochondria-assisted diseases, aging, neurodegenerative diseases and other associated pathologies, hormesis is found to be an integral part of many of the contributing cell signaling pathways and systems. Since discussing hormesis in all stressor-mediated pathways that promote disease is beyond the scope of a single article, we have chosen only a few representative pathways that are modulated by hormesis and are simultaneously implicated in different diseases, such as cardiovascular diseases, aging, neurodegenerative problems and others.

### 4.1. Endoplasmic Reticulum Stress

**Figure 2:**
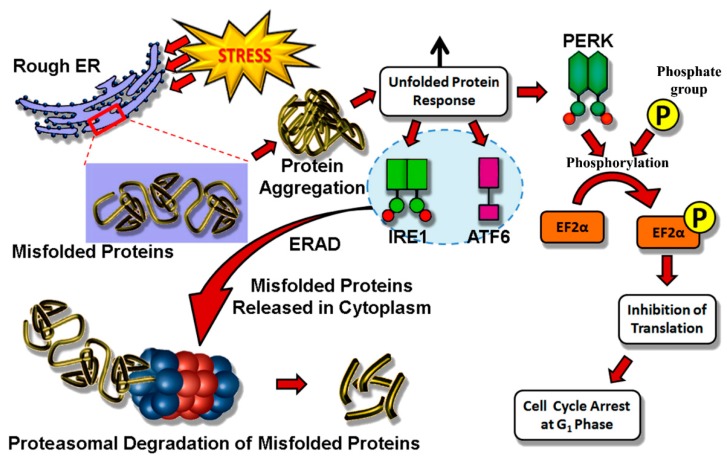
Molecular basis of the endoplasmic reticulum stress mechanism. Accumulation of misfolded proteins leads to upregulation of unfolded protein response (UPR) activating three pathways mediated by IRE1, ATF6 and PERK, respectively. Endoplasmic reticulum-associated degradation (ERAD) pulls out misfolded proteins into the cytoplasm and induces proteasomal degradation.

Several factors, such as calcium depletion, ROS and mutations in trafficking proteins of the secretory pathway, can generate ER stress. This is characterized by the aggregation of misfolded proteins, which trigger an unfolded protein response (UPR). ER-hormesis was studied for the first time in *Drosophila* ninaA chaperone mutants where accumulation of misfolded Rhodopsin-1 was observed to induce an adaptive UPR [60]. The latter is a conserved adaptive response that activates three pathways (namely inositol-requiring enzyme 1 (IRE1), protein kinase RNA-like ER kinase (PERK) and activating transcription factor 6 (ATF6)) on sensing disruption to normal ER physiology [61]. IRE1 and ATF6 pathways elicit ER-associated degradation (ERAD) that relocates misfolded proteins from ER to cytoplasm, so that the erroneously-folded proteins can undergo proteasomal degradation [62]. However, PERK phosphorylates elongation factor 2α (EF2α) that hinders translation of nascent proteins (Figure 2). Interestingly, cell survival/death is determined by the hormetic role of ER-mediated stress. While a prolonged, strong UPR activation mediates apoptosis by activating DNA damage-inducible transcript 3 (DDIT3), mild stress renders protection in neurodegenerative disease models. Another study in *Drosophila* and mice models of Parkinson’s disease revealed that mild UPR activation could promote autophagy-assisted neuroprotection [63]. From studies involving *Drosophila* and mice, it is concluded that cellular protection is rendered by the activation of X-box binding protein 1 (XBP1) [61]. In addition, ER hormesis finds special significance as a plausible therapeutic target in cancer intervention. Mutations in G-protein-coupled receptor smoothened (Smo), a member of the hedgehog signaling pathway, is associated with cancer manifestation [61]. This particular protein is clinically relevant since it is closely associated with resistance in chemotherapy. Marada and co-workers have reported that ERAD-specific ubiquitin (Hrd1) could successfully destabilize Smo-mutants through UPR. Interestingly, this destabilization is selectively targeted to mutants without affecting the wild-type *D. melanogaster* [64].

### 4.2. Mitochondria and ROS

Accumulation of ROS-induced oxidative damage in mtDNA has been earmarked to be a major contributor to aging [65]. Mitochondrial dysfunction is associated with large-scale production of ROS that ultimately promotes cellular degradation [66]. For example, arsenite, a notorious toxin, is known to alter mitochondrial homeostasis (via ROS production and electron transfer chain disruption) [67]. However, administering low doses of arsenite in *C. elegans* was found to promote longevity [68]. The mechanism behind this biphasic behavior of arsenite was conferred to its ability to activate transcription factors SKN-1 and DAF-16 (orthologs of mammalian Nrf2 and FOXO) [68].

### 4.3. Insulin or Insulin-Like Growth Factor 1 Signaling

Insulin and IGF-1 are produced in pancreas and liver, respectively, and the latter’s release is regulated by growth hormone. While impairment of insulin/IGF-1 leads to reduced growth in mice, it simultaneously advocates longevity and protection from neurodegeneration. Similar results were obtained in *C. elegans* and *Drosophila* models, as well [69,70]. A large-scale cohort study in humans revealed that polymorphisms in FOXO3A and AKT1 (genes associated with insulin/IGF-1) were markers of increased life span [71].

Thus, hormesis plays a crucial role in the manifestation of a myriad number of diseases. Furthermore, the same biphasic trait enables the onset of beneficial activities in the ‘diseased’ individual. In the next section, we discuss the role of hormesis in the context of cancer.

## 5. Hormesis and Cancer: A Potential Rescue Path

Cancer is one of the leading causes of death worldwide, which shows an alarming rate of incidence [72]. It is estimated that by 2030, the number of cancer patients might increase by almost 21 million [73]. While humongous chunks of money are being pushed into the area of cancer research, the complexities of pathways and their cross-talk still obscure any comprehensive mode(s) of cure. Although a number of chemotherapeutic reagents have definitely given a boost in managing the disease, more specific therapeutics are still needed [15]. Moreover, the fact that many research articles have cited the dose-dependent response of the available therapeutics, it becomes all the more pragmatic for regulatory bodies to delve into the possibility of the hormetic effects of the drug [15]. In a recent work carried out by Pearce and co-workers, immune reactants (cancer antibodies) demonstrated alternate stimulatory and inhibitory effects on tumor growth for low and high doses, respectively [74]. Similarly, lithium chloride was also found to be hormetic towards inhibition/induction of apoptosis in breast cancer cells [75]. Herein, we discuss a few critical pathways that play pivotal roles in cancer progression and also act as a seat of hormesis. Since it is impossible to assimilate all pathways that have functional duality associated with them, we categorically look into a few common pathways associated with hormesis.

### 5.1. Nrf2-Keap1 Signaling Pathway

Nrf2 is a transcription factor that co-ordinates the upregulation of around 200 genes associated with diverse cytoprotective mechanisms [76]. During non-stress conditions, the half-life of Nrf2 remains short, and its levels are regulated by Keap1 (both form a complex together in the cytoplasm) [77]. Additionally, polyubiquitination and proteasomal degradation of Nrf2 is carried out by the Keap1-Cullin 3/RING-box protein 1 (Rbx1)-E3 ligase complex, ensuring its sequestration in the cytoplasm [78]. However, on events of stress, conformational changes in Keap1 inhibit ubiquitination and subsequent degradation of Nrf2. This allows translocation of the latter into nucleus, where it binds to ARE/EpRE (antioxidant/electrophile response element) eliciting expression of cytoprotective molecules. Interestingly, the duality of Nrf2 lies in the fact that in the case of a carcinogenic stress, Nrf2 mediates the adaptive response through the expression of phase II detoxifying enzymes, rendering protection to cells against carcinogen-mediated genotoxicity [79]. On the contrary, in diverse types of cancer, loss of function of Keap1 was observed to assure prolonged activation of Nrf2, thereby upregulating its downstream genes and subsequently facilitating the growth of neoplastic cells [79,80,81]. Furthermore, p21 and p62 can bind to Nrf2 and Keap1, respectively, thus blocking Nrf2-Keap1 complex formation and subsequent monitoring of Nrf2 levels by Keap1 [82].

### 5.2. NFκB Signaling Pathway

NFκB is a transcription factor that forms an inactive cytosolic heterodimer with p65(RelA), RelB and others. This complex stays in association with inhibitors, such as IκB, BCL-3, *etc.* Stressor (ROS, cytokines, tumor necrosis factor (TNF), Toll-like receptors (TLRs), *etc.*) mediating the removal of these inhibitors assure activation and subsequent nuclear translocation of NFκB [30]. Usually, inflammatory response is an outcome of NFκB activation highlighted by the upregulation of TNFα, IL-2/-6, intercellular adhesion molecule 1 (ICAM1), vascular cell adhesion molecule 1 (VCAM1) proteins, cyclooxygenase 2 (COX-2) and inducible nitric oxide synthase (iNOS)-producing enzymes [83]. This is of particular interest, because several proteins upregulated due to NFκB activation are actually NFκB activators. Thus, they initiate an amplified initial response sustaining inflammation, leading to safety against an external/internal stressor. However, the other side of this pathway is the upregulation of certain proteins that inhibit autophagy or apoptosis. Therefore, on the one hand, while NFκB defends cells from stressor by generating inflammation, the same factor is responsible for promoting survival and growth in cancer cells through the induction of the inhibitor of apoptosis proteins (IAPs) and TNFα, respectively [83,84]. This builds up a nexus between inflammation and cancer, with NFκB being the epicenter of hormesis.

### 5.3. Sirtuin-FOXO Signaling Pathway

SIRTs are nicotinamide adenine dinucleotide (NAD^+^)-dependent protein deacetylases that cater to a wide range of cellular processes in response to stress [85]. Mammalian sirtuins constitute seven members (SIRT1-7) that vary hugely in terms of their cellular localization and functionality [86]. This family of deacetylases shows a hormetic response to stress, particularly in cancer. Some like SIRT1 confer protection by guarding DNA from stress-mediated damage and oncogene (PML/p53)-induced senescence preventing cancer [87]. SIRT1 has also been observed to promote tumor growth and overall survival and progression of cancer cells [88]. One of the major pathways in cancer progression involves sirtuin and the transcription factor FOXO. FOXO (acts downstream of tumor suppressor phosphatase and tensin homolog (PTEN)) on activation (by SIRT1-mediated deacetylation) translocates into nucleus and upregulates genes associated with DNA repair, cell cycle arrest and apoptosis. However, in prostate cancer cells, deacetylation of FOXO1 by SIRT1 was found to hinder the former’s transcriptional and apoptotic activities [89]. This hormetic behavior of tumor promotion/suppression by sirtuins based on the differences in the associated targets and signaling pathways makes them an interesting target in cancer.

## 6. Hormetic Compounds in Cancer

Duality is a trait restricted not only to pathways, but is prevalent among compounds, as well. There are several compounds (natural, as well as synthetic) that, owing to their hormetic property, confer protection to normal cells, as well as are capable of inducing signaling cascades that lead to apoptosis of cancerous cells. In other words, there is a plethora of compounds that inflict different activities in diverse cellular systems owing to the variation in the doses of administration. We take a glance at a few such compounds and their brief *modi operandi*, particularly under the realms of probable anti-cancer therapeutics (Table 1).

This table is a mere compilation of very few, prominent compounds relevant to cancer therapeutics. As the arena of hormesis is comparatively new (since its re-invention in the 1970s), more impetus needs to be directed towards finding out the biphasic dose response of all drugs to be tested or those that are already commercially available. This information would be a tremendous asset, since the duality in the same compound/pathway can be employed to devise innovative flexible strategies to curb pertinent problems of disease manifestation and progression.

**Table 1:** Hormetic compounds with cytotoxic activity towards cancer cells *in vitro* and *in vivo*.

Compound	Low Dose	High Dose	Plausible Mode of Mechanism	Cancer
ATN-161	↓angiogenesis	↑cytotoxic	Integrin inhibitor [90]	Colon
Chalcone	non-toxic	cytotoxic	Nrf2 activation; inhibits NFκB; TRAIL-mediated hypoxia-induced apoptosis [91]	Ovarian, hepatic
Cilengitide	↑angiogenesis	↓angiogenesis	Inhibits αvβ3 and αvβ5 integrins; endothelial cell migration [92]	Subcutaneous tumor graft
Curcumin	↑HO-1, neuro-protection	↑DNA damage, apoptosis	Reduces matrix metalloproteinases’ expression through downregulation of NFκB and AP-1 [93,94]	Breast
Dithiolethione	neuro-protection	cytotoxic	Increases Nrf2/ARE pathway-mediated transcriptional activity of phase II enzymes [66]	-
Endostatin	↓angiogenesis	cytotoxic	Inhibits endothelial cell proliferation, migration [95]	Pancreatic
Epigallocatechin	neuro-protection	pro-oxidant, ↑apoptosis	Phosphorylates Bad at Ser-112,136 through ERK, AKT pathways; Bcl-2:Bax increases [96]	Neuroblastoma
Genistein	↑proliferation	↓proliferation	Increases cleaved PARP expression; inhibits NFκB; inhibits Akt [97]	Prostate
Isothiocyanates	↑proliferation	↓proliferation	Alters cell growth and migration pattern [98]	Colon
Kaempferol	estrogen agonist	growth inhibitor	Depletes estrogen-induced malignancy; suppresses COX-2; induces caspase-3, apoptosis inducing factor (AIF), MnSOD [99]	Breast
Metformin	anti-diabetic	anti-cancer	Suppresses mTOR/S6K1; inhibits tyrosine kinase receptors HER1/2 [100]	Epidermoid, breast, prostate
Quercetin	anti-oxidant	pro-oxidant	Suppresses NFκB activity, G1 cell cycle arrest, ↑p21, p53; inhibits ubiquitination [15]	Pancreatic, colon, hepatic
Resveratrol	↑proliferation cardio protection	↓proliferation anti-cancer	Activates Nrf2; upregulates FOXO [101,102]	endometrial
Rosiglitazone	↓angiogenesis	↑cytotoxicity	Inhibits endothelial proliferation and vascular endothelial growth factor (VEGF) activation; upregulates matrix metalloproteinase (MMP) inhibitors [103]	Bladder, breast, thyroid
Secoiridoid	↓pro-aging effect	↑cytotoxicity	Activates ER stress, unfolded protein-mediated response, SIRT1 and Nrf2 [21]	Breast
Sulforaphane	↑proliferation ↑angiogenesis	↓proliferation ↓angiogenesis	Activates Nrf2/ARE pathway; regulates NFκB and AP-1 to induce apoptosis; activates autophagy [98]	Bladder
Thrombo-spondin-1	↑cell migration	↓cell migration	Inhibits endothelial cell migration. [104]	Oral

↑: increase; ↓: decrease.

## 7. Hormesis Mimetics: A World of Endless Opportunities

Mimesis (to imitate) in research is a powerful tool, which, if applied judiciously, can work wonders, particularly in understanding disease mechanisms. The compounds/factors that mimic a known factor/pathway/compound are often referred to as mimetics. In the field of hormesis, mimetics are agents that can induce hormetic pathways [35]. Exposure to such agents ensures protective responses, not only to the imminent threat, but may also reverse the functionality errors associated with age/stress/disease to a certain extent [105]. However, it must be remembered that the dose/concentration of the mimetic being administered should itself not be toxic to the body and must be provided in moderate (harmless) quantities. Based on the type of hormetic pathway accessed and the factors elicited/inhibited to promote a response, hormesis mimetics can be broadly categorized as follows.

### 7.1. Heat Mimetics

These enhance HSP response to heat stress through the activation of chaperones, leading to lesser aggregation of misfolded proteins. This, in turn, assists in the proteasomal degradation of anomalous proteins subsequently equipping the cell/system for future stresses [35].

### 7.2. CR Mimetics

CR mimetics are molecules that are the biochemical and functional facets of CR. They promote deacetylation of cellular proteins and mediate autophagy. In addition, properties, such as glycolysis inhibition, sirtuin, lipid regulation and insulin-specific gene modulation, enable this group of mimetics to promote longevity, anti-aging and anti-cancer activities [106,107].

### 7.3. Radiation Mimetics

This type of molecule stimulates the resistance pathways of the DNA repair of radiation-induced damage. They also trigger the signaling pathway associated with the gene cascade whose expression ensues longevity and protection from radiation-mediated injuries [108].

### 7.4. Hibernation Mimetics

These cater to a stratagem similar to that of hibernation, which forms a significant policy of evading stress-induced damage. Hibernation is a common phenomenon that is adopted to tackle the problems of hypoxia, hypothermia, intracellular acidosis or muscle wasting. Such agents help in the mitigation of multiple stresses [35].

Apart from the broad categories mentioned above, exercise and immunoregulatory mimetics enhance the overall protective response of an individual exposed to stress. In Table 2, we have enlisted a few mimetics that are common in the field of hormesis and are associated with disease alleviation to a considerable extent.

**Table 2:** Hibernation mimetics associated with disease alleviation.

Compound	Type of Mimetic	Plausible Mode of Mechanism	Disease
Carnitine	caloric restriction	Upregulates HO-1, sirtuin, thioredoxin, ↓pro-oxidant activity, mediates fatty acid metabolism [66]	Neurodegenerative damage
Resveratrol	caloric restriction	Sirtuin activator, ↓UV/H_2_O_2_-induced apoptosis, ↑SIRT mediated FOXO activation [109,110]	Longevity, oxidative damage, toxicity resistance
Secoiridoid	caloric restriction	↑Nrf2, SIRT1 signaling, mediates ER stress response, regulates mTOR pathway [21]	Longevity, age-associated diseases
PPARδ agonists	caloric restriction	↓Glucose consumption in skeletal muscles [111]	Insulin sensitivity
Ethanol	heat	↑Hsp70 in brain; ↓β amyloid-induced neurotoxicity and apoptosis [112]	Alzheimer’s; dementia
Geranyl-geranylactone (GGA)	heat	Induces bone osteoblasts, upregulates thioredoxin, forms apoptosome on binding to Apaf-1, inhibits c-Jun N-terminal kinase [113,35]	Osteoporosis, increases immunity, apoptosis in normal cells
Delta 2 opioid receptors (DADLE)	hibernation	↓Neuronal damage. [114]	Neurodegeneration
Oltipraz	radiation	↑Nrf2-ARE binding, ↑transcriptional induction of carcinogen detoxification gene cascade [115]	Oxidative stress, cancer
Ferritins	radiation	ARE activation, ↓ROS-mediated damage [35]	Oxidative stress
Thiols and metals	radiation	↑Antioxidant gene expression, ↑glutathione peroxidase activity [116,117]	Radiation induced oxidative damage, cancer
Oligonucleotides	radiation	↓Mutagenesis, photo-carcinogenesis, ↑DNA repair, mitochondria hyperpolarization [108,118]	UV-induced mutation, cancer
Conserved peptide sequences, CpG oligo		↑TH2-mediated inflammation, ↓cytokine dysfunction, ↑adaptive immunity [35]	Increases immunity

↑: increase; ↓: decrease.

## 8. Conclusions

Now that hormesis is gaining prominence in the context of its imperative relevance in the field of biology, it becomes absolutely mandatory to determine the biphasic activities of therapeutics, or pathways, or stressors. It is known from a number of reports that early life epigenetic alterations can impose a long-term effect on genetic and phenotypic expression. Mild stress-mediated hormesis promotes epigenetic adaptations, and the subsequent transcriptional re-organization forms the basis of all hormetic mechanisms [119]. Understanding how hormesis mechanistically governs life processes would unfurl the mystery towards accurately determining the suitable dosages, suitable signaling molecules or maybe suitable agents that can mimic the effect of an otherwise hormetic substance. These would further help in identifying targets and devising effective strategies to ameliorate diseases or their associated complications. After all, complete knowledge about both sides of a coin definitely tells us much more about the coin.
